# Long term costs and effects of reducing the number of twin pregnancies in IVF by single embryo transfer: the TwinSing study

**DOI:** 10.1186/1471-2431-10-75

**Published:** 2010-10-20

**Authors:** Mirjam MJ van Heesch, Gouke J Bonsel, John CM Dumoulin, Johannes LH Evers, Mark AHBM van der Hoeven, Johan L Severens, Ramon HM Dykgraaf, Fulco van der Veen, Nino Tonch, Willianne LDM Nelen, Piet van Zonneveld, Johannes B van Goudoever, Pieter Tamminga, Katerina Steiner, Corine Koopman-Esseboom, Catharina EM van Beijsterveldt, Dorret I Boomsma, Diana Snellen, Carmen D Dirksen

**Affiliations:** 1 Department of Clinical Epidemiology and Medical Technology Assessment, Maastricht University Medical Centre, P.O. Box 5800, 6202 AZ Maastricht, The Netherlands; 2 Department of Obstetrics & Prenatal Medicine, Room he-113, Erasmus Medical Centre, P.O. Box 2040, 3000 CA Rotterdam, The Netherlands; 3 Midwifery Academy Rotterdam, building "Rochussenstraat" GK-754, P.O. Box 2040, 3000 CA Rotterdam, The Netherlands; 4 Department of Public Health, Erasmus Medical Centre, P.O. Box 2040, 3000 CA Rotterdam, The Netherlands; 5 Department of Obstetrics and Gynaecology, GROW, School for Oncology and Developmental Biology and Maastricht University Medical Centre, P.O. Box 5800, 6202 AZ Maastricht, The Netherlands; 6 Department of Neonatology, Maastricht University Medical Centre, P.O. Box 5800, 6202 AZ Maastricht, The Netherlands; 7 Department of Health Organisation, Policy, and Economics, Maastricht University, P.O. Box 616, 6200 MD, Maastricht, The Netherlands; 8 Institute of Health Policy and Management, Erasmus University Rotterdam, P.O. Box 1738 3000 DR Rotterdam, The Netherlands; 9 Department of Obstetrics and Gynaecology, Division of Reproductive Medicine, Erasmus Medical Centre, s´Gravendijkwal 230, 3015 CE Rotterdam, The Netherlands; 10 Centre for Reproductive Medicine, Academic Medical Centre, P.O. Box 22660, 1100 DD Amsterdam, The Netherlands; 11 Department of Obstetrics and Gynaecology, Radboud University Nijmegen Medical Centre, P.O. Box 9101, 6500 HB Nijmegen, The Netherlands; 12 Department of Reproductive Medicine, Division of Obstetrics, Neonatology and Gynaecology, University Medical Center Utrecht, P.O. Box 85500, 3508 GA Utrecht, The Netherlands; 13 Department of Pediatrics, Emma Children's Hospital, Academic Medical Centre, P.O. Box 22700, 1100 DD Amsterdam, The Netherlands; 14 Department of Pediatrics, VU University Medical Center, P.O. Box 7057, 1007 MB Amsterdam, The Netherlands; 15 Department of Pediatrics, Sophia Children's Hospital, P.O. Box 2060, 3000 CB Rotterdam, The Netherlands; 16 Department of Neonatology, Academic Medical Centre, Emma Children's Hospital, P.O. Box 22700, 1100 DD Amsterdam, The Netherlands; 17 Department of Neonatology, Radboud University Nijmegen Medical Centre, P.O. Box 9101, 6500 HB Nijmegen, The Netherlands; 18 Department of Neonatology, Wilhelmina Children's Hospital, University Medical Center Utrecht, P.O. Box 85090, 3508 AB Utrecht, The Netherlands; 19 Department of Biological Psychology, VU University Amsterdam, Van der Boechorststraat 1, 1081 BT Amsterdam, The Netherlands; 20 Freya, Association for people with fertility problems, P.O. Box 476, 6600 AL, Wijchen, The Netherlands

## Abstract

**Background:**

Pregnancies induced by in vitro fertilisation (IVF) often result in twin gestations, which are associated with both maternal and perinatal complications. An effective way to reduce the number of IVF twin pregnancies is to decrease the number of embryos transferred from two to one. The interpretation of current studies is limited because they used live birth as outcome measure and because they applied limited time horizons. So far, research on long-term outcomes of IVF twins and singletons is scarce and inconclusive. The objective of this study is to investigate the short (1-year) and long-term (5 and 18-year) costs and health outcomes of IVF singleton and twin children and to consider these in estimating the cost-effectiveness of single embryo transfer compared with double embryo transfer, from a societal and a healthcare perspective.

**Methods/Design:**

A multi-centre cohort study will be performed, in which IVF singletons and IVF twin children born between 2003 and 2005 of whom parents received IVF treatment in one of the five participating Dutch IVF centres, will be compared. Data collection will focus on children at risk of health problems and children in whom health problems actually occurred. First year of life data will be collected in approximately 1,278 children (619 singletons and 659 twin children). Data up to the fifth year of life will be collected in approximately 488 children (200 singletons and 288 twin children). Outcome measures are health status, health-related quality of life and costs. Data will be obtained from hospital information systems, a parent questionnaire and existing registries. Furthermore, a prognostic model will be developed that reflects the short and long-term costs and health outcomes of IVF singleton and twin children. This model will be linked to a Markov model of the short-term cost-effectiveness of single embryo transfer strategies versus double embryo transfer strategies to enable the calculation of the long-term cost-effectiveness.

**Discussion:**

This is, to our knowledge, the first study that investigates the long-term costs and health outcomes of IVF singleton and twin children and the long-term cost-effectiveness of single embryo transfer strategies versus double embryo transfer strategies.

## Background

In the Netherlands, about 16,000 IVF treatments are performed every year [1]. As a result, approximately 2.3% of all Dutch children are born from a pregnancy established by in vitro fertilization (IVF) [2]. A common complication of IVF is multiple pregnancy [3], which occurs in 20-25% of all IVF pregnancies [4-7]. Obstetricians and neonatologists express a preference to prevent twin pregnancies in IVF because of maternal and perinatal complications [7]. Pregnancy-induced maternal complications occur three to seven times more often in multiple than in singletons pregnancies [8]. The most common maternal complications of a twin pregnancy are pregnancy-induced hypertension, pre-eclampsia, anaemia, antepartum and postpartum haemorrhage, uterine atony and dystocia, increased operative delivery, uterine rupture and preterm labour [8,9]. It is well known that twin status is associated with perinatal complications. Perinatal morbidity and mortality is increased four-to ten-fold in twins [8]. Twins are born prematurely more often than singletons. Approximately 50% of twin deliveries occur before 37 weeks gestation [10-12], and this is considered to be the factor that is largely responsible for the excess of perinatal and neonatal deaths and neonatal morbidity in twins [11,13,14]. Twins also have a lower birth weight compared to singletons [9,15,16], even when born full term [17]. Because of the high frequency of complications, the average twin pregnancy requires more medical care than a singleton pregnancy, resulting in higher costs. Results from the Dutch TWIN study indicated that hospital costs of children born from IVF up to one year following birth add up to about €3,000 for singletons and €9,000 for twin children [18].

The explanation for the increased risk of a multiple pregnancy after IVF is the policy of transferring two embryos into the uterus (double embryo transfer; DET) [19], which is still customary in the majority of women receiving IVF treatment, particularly in older women. A successful way to reduce the number of twin pregnancies after IVF is to transfer only one embryo (single embryo transfer; SET) [20-26]. The current policy in The Netherlands is to offer SET in good prognosis patients (i.e. young patients with good quality embryos). The decision for the number of embryos to transfer - either one or two embryos - is established by both the couple and professionals [27].

Several short-term (cost-) effectiveness studies have shown that transferring one fresh embryo and then, if needed, one frozen-and-thawed embryo dramatically reduces the number of twin pregnancies while achieving similar cumulative pregnancy rates to transferring two embryos in good prognosis patients [20,21,23-26,28-30]. A review by Fiddelers *et al*. [31] of economic evaluations of SET versus DET concluded that, from a cost-effectiveness point of view, SET is only preferred in good prognosis patients and when frozen-and-thawed cycles are included. The conclusions of the review are confirmed by a detailed (cost-)effectiveness analysis [20,26] and a Markov cost-effectiveness model [32]. The Markov model compared seven embryo transfer strategies varying from three cycles SET to three cycles DET and several combination strategies. From the Markov-chain based study, it was concluded that DET is more effective but also more costly compared to SET, with an Incremental Cost-Effectiveness Ratio (ICER) around €20,000 for an extra live birth.

Interpretation of current cost-effectiveness studies comparing SET strategies with DET strategies is complicated. First, *live birth *is frequently used as outcome measure. To reflect the fact that twin pregnancies and twin births carry a higher risk for complications than singleton pregnancies and singleton births, both singleton and twin births are counted as one unit of live birth. However, based on results of preference studies, counting a twin birth as one live birth is incorrect from a subfertile couples' point of view [33-39]. Furthermore, a live birth is an intermediate outcome as long-term consequences are not included. Besides, it is not a preference-based outcome measure as opposed to Quality Adjusted Life Years (QALYs), which is a commonly used outcome measure in economic evaluations. Second, published cost-effectiveness analyses applied a limited time horizon, up to six months after delivery. If a child experiences permanent morbidity the consequences are lifelong, and thus data on long-term costs and outcomes associated with singletons and twins born after IVF should be included in the analysis in order to obtain a valid estimate of the incremental cost-effectiveness of different embryo transfer strategies. Third, most parents prefer a complete family. This implies that cost-effectiveness studies should preferably include the first IVF treatment as well as subsequent attempts for a second child. Fourth, even with current data, a societal preference statement on the choice between SET and DET depends on the amount of money society is prepared to pay for one extra live birth (ceiling ratio) [31]. No agreement exists on an appropriate ceiling ratio for one extra live birth, as opposed to the ceiling ratio for a QALY [1,40]. The right to procreate is generally regarded as fundamental, and from this principle one would expect a high ceiling ratio.

The proposed study - the TwinSing study - will investigate the short (1-year) and long-term (5 and 18-year) costs, health outcomes and health-related quality of life (HRQoL) of twins and singletons born from IVF for broad use in cost-effectiveness analyses comparing SET strategies with DET strategies.

### Current evidence

Most studies on the long-term follow up of IVF children so far did *not *compare IVF twin children with IVF singletons, but compared children born from IVF with naturally conceived children [41-45].

#### Long-term health-related outcomes

Up till now only few studies have actually compared long-term health-related outcomes of IVF twins and IVF singletons [46-48]. They reported varying results regarding physical health and developmental outcomes. Pinborg et al. [48] reported that twins between two and seven years of age have a similar risk of neurological sequelae and cerebral palsy compared to singletons of the same age. In another study, Pinborg et al. [47] observed no discrepancies between IVF twins and IVF singletons aged three to four years regarding severe neurological disabilities, allergic disorders, common infections and motor function. Nevertheless, IVF twins had poorer speech development and poorer general health status. Mortality rates did not differ significantly between IVF twins and IVF singletons, although there was a tendency towards a higher mortality rate in IVF twins. Bonduelle et al. [46] reported that the occurrence of malformations was not statistically different between IVF twins and singletons, whereas the developmental outcomes of IVF twins, as measured with the Bayley instrument, were significantly worse compared to IVF singletons at the age of two years.

Other studies compared both IVF singletons with naturally conceived singletons and IVF twins with naturally conceived twins [42-45]. Although these studies provided figures about the long-term health status of IVF twins and IVF singletons, no statistics were presented and no conclusions were drawn regarding the comparison between IVF twins and IVF singletons. All three studies suggested that the health of IVF twins was worse than that of IVF singletons. Klemetti et al. [43] showed that the risks for cerebral palsy, behavioural disorders, asthma, pneumonia and diarrhoea were higher for IVF twins aged two years compared with IVF singletons of the same age. Koivurova et al. [44] reported that six out of eight diseases were more prevalent in IVF twins than in IVF singletons, i.e. respiratory diseases, pneumonia, obstructive bronchitis, asthma, juvenile arthritis and neurological signs. Another study of Koivurova et al. [42] showed that risks of having diagnoses related to diseases affecting the central nervous system were higher for IVF twins. Furthermore, the proportion of children with low weight, low height and inability to perform at least one developmental test was higher in the IVF twin group compared to singletons for several age categories up to three years. Strömberg et al. [45] found a higher proportion of neurological sequelae in IVF twins compared to IVF singletons aged 18 months to 14 years. Overall, the results of these studies are ambivalent, some hinting at worse outcomes for IVF twins and some showing similar outcomes for IVF twins and singletons.

#### Long-term costs

At this moment only a few cost studies have been performed [41-43,47,49]. Most studies were restricted to an evaluation of the use of hospital resources. Two studies statistically compared long-term resource use and/or costs of IVF twins and IVF singletons [47,49]. A study by Pinborg et al. [47] revealed no difference in the risk of hospitalization and the number of hospitalizations per child between IVF twins and IVF singletons aged three to four years. In contrast, they showed in another study that IVF twins aged two to seven years were more likely to be admitted to a hospital, had more admissions per child, longer hospital stays, as well as more surgical interventions and special needs [49]. Even term twins were more likely to be hospitalized than term singletons [49].

Other studies did not statistically confirm differences in resource use or costs of IVF twins and IVF singletons [41-43]. Koivurova et al. [42] concluded that the costs of post-neonatal care were almost equal for IVF twins and IVF singletons during a seven-year follow-up period. A large registry study by Ericson et al. [41] with an average follow-up of six years showed that IVF twins had about twice as much hospital days as IVF singletons. Klemetti et al. [43] also reported more resource use by IVF twins up to two to four years of age. Medication use was measured up to two years of age, whereas the use of hospital services was measured up to four years of age. More IVF twins than IVF singletons had long-term medication use, were hospitalised, and had longer hospital stays. Overall, the majority of these studies reported higher use of resources for IVF twins. It has been estimated that lifetime extra health care costs of a twin pregnancy add up to €30,000 in the Netherlands [50].

#### Long-term impact on parent's life

Mothers of two to five year old IVF twins experienced significantly higher levels of parenting stress and depression than mothers of IVF singletons and were less likely to obtain pleasure from their child and to be in paid employment [13,51].

#### Conclusion

Overall the current evidence confirms that post-neonatal costs are higher in IVF twins compared to IVF singletons. Reported results regarding health outcomes are inconclusive. Some studies were, however, not designed to detect significant differences between IVF twins and IVF singletons and probably lacked statistical power to detect differences. The available evidence is insufficient to estimate the long-term cost-effectiveness of SET strategies compared with DET strategies.

### Problem definition

In the Netherlands, long-term results from IVF children are currently lacking. It is timely to obtain an objective estimate of the long-term costs and health outcomes of twins and singletons born from IVF, to support the claim that twin pregnancies are a complication of IVF that should be prevented. Objective here means empirically based and taking account for sources of bias. To support the general believe that twin pregnancies are a complication of IVF and to make an evidence-based decision on the preferred embryo transfer strategy in IVF, both long-term costs and health outcomes of twins and singletons need to be estimated.

The TwinSing study is funded by the Netherlands Organisation for Health Research and Development (ZonMW), grant number 80-82310-98-09094. The study is approved by the Institutional Ethical Board of the Maastricht University Medical Centre (Ref no: 09-4-019) and the study is actively supported by the Dutch patient association Freya. The Ethical Board of the Maastricht University Medical Centre judged that the TwinSing study is not subject to the Medical Research Involving Human Subjects Act (WMO).

### Objective and research questions

The objective of the TwinSing study is to investigate the short (1-year) and long-term (5 and 18-year) costs, health outcomes and HRQoL of twins and singletons born from IVF. These results will be linked to a Markov model in which seven embryo transfer strategies are compared [32], to enable estimation of the long-term cost-effectiveness of SET strategies versus DET strategies.

The research questions are:

1. What are the short (1-year) and long-term (5 and 18-year) health outcomes of IVF twins and singletons?

2. What are the short (1-year) and long-term (5 and 18-year) costs of IVF twins and singletons?

3. What is the short (1-year) and long-term (5 and 18-year) cost-effectiveness of SET strategies versus DET strategies from a societal and healthcare perspective?

4. What is the value of additional information through research to reduce decision uncertainty?

## Methods/Design

### Design

A retrospective multi-centre (n = 5) cohort study will be performed, in which a representative sample of IVF twin children will be compared with a representative sample of IVF singletons with respect to mortality, health outcomes, HRQoL and costs from birth up to five years of life. Subsequently, future costs and health outcomes up to 18 years of life will be modelled. Input for the model will be based on the empirically collected data up to five years of life, existing databases, literature and expert opinion.

### Participants

The study population consists of twins and singletons born from IVF between 2003 and 2005, of whom parents have received IVF treatment in one of the five participating IVF centres in the Netherlands (Academic Medical Centre, Maastricht University Medical Centre, Radboud University Nijmegen Medical Centre, Erasmus Medical Centre and University Medical Center Utrecht). In the Netherlands, subfertility care is well-organized and centred in thirteen licensed clinics [52]. Hospitals without a licence can initiate and monitor the stimulation phase and refer to a licensed hospital for both oocyte retrieval and embryo transfer (satellite clinics) or for embryo transfer alone (transport clinics). Five of the 13 licensed centres and their collaborating transport- and satellite clinics, participate in the TwinSing study.

The *full sample *consists of all IVF twins and IVF singletons born between 2003 and 2005 of whom parents have received treatment in one of the participating centres. A total of 4,809 ongoing pregnancies were reported by these IVF centres between 2003 and 2005, of which - 3,789 singleton and 1,020 twin pregnancies. The actual size of the full sample will be somewhat lower since the reported figures are indicative; they are based on ongoing pregnancies not live births. Inclusion of an older cohort of IVF children is not considered to be valid, since the quality and results of IVF, neonatal and paediatric care have improved considerably during the last decade and since this study relies heavily on retrospective data collection, both from existing registries as well as from parents.

**Figure 1:**
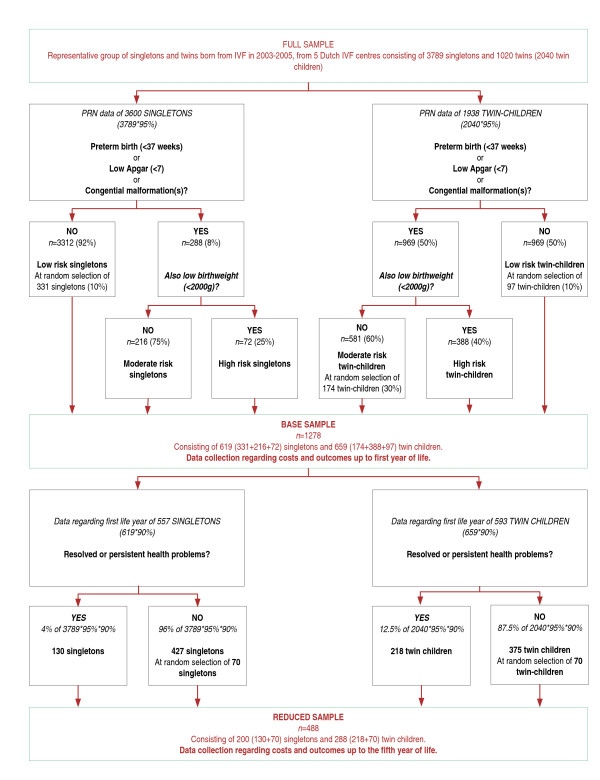
**Risk stratification and sample size (figures are indicative)**.

Data collection will primarily focus on the subgroup of children 'at risk' for health problems and on those children in which health problems have actually occurred. Therefore, for all children included in the full sample, information from the Perinatal Registry Netherlands (PRN) will be studied. Based on information from the PRN and the establishment of a clear set of criteria, all children at 'high risk' and a random sample of children at 'low risk' will be selected. All singleton children at 'moderate risk' and a random sample of twin children at 'moderate risk' will also be selected (see figure 1). Together, these children will form the basis for empirical data collection with respect to the first year of life, and will further be referred to as the *base sample*. Based on results up to the first year of life, the base sample of children will further be reduced for detailed data collection regarding the costs and health outcomes of the children up to five years of life and data estimation up to 18 years of life (further referred to as the *reduced sample*). Infant will be the unit of analysis. The selection of IVF twin children of a pair for inclusion in the base sample and reduced sample will occur independently.

In order to sufficiently take account of heterogeneity within the children who experienced health problems, it is expected that costs and outcome data of at least 100 singletons and 100 twin children with health problems are required in the reduced sample. Regarding children without health problems, data of 50 singletons and 50 twin children is expected to be sufficient, as variance will be considerably lower. Taking sufficiently account of missing data in all phases of data collection (5% for risk stratification, 10% for data collection up to one year of life and 20% for data collection up to five years of life), a sample size of 175 twin children with health problems, 56 twin children without health problems, 104 singletons with health problems, and 56 singletons without health problems, is required for the reduced sample.

### Data collection

#### Data collection full sample

The five participating IVF centres will each provide a dataset consisting of mothers who have had an effective IVF treatment and gave birth to a singleton or twin between 2003 and 2005. The data received from the IVF centres will be linked to the PRN database, which encompasses information regarding mother, pregnancy and newborn. The data will be matched to the PRN database based on the birth date of the mother, parity of the mother, (an estimation of) the birth date of the child(ren), and whether it was a singleton or twin birth.

#### Risk stratification

Risk stratification will be applied to all children in the full sample. Based on information from the PRN regarding the pregnancy (weeks gestation) and the newborn (birth weight, Apgar score and congenital malformations), the children of the full sample will be assigned to a risk profile (i.e. 'low risk', 'moderate risk' or 'high risk'). The risk stratification criteria are: 1) prematurity (< 37 weeks gestation); 2) 5-minute Apgar score (< 7); 3) congenital malformations; and 4) birth weight (< 2,000 gram) (see figure 1). The criteria are set such that the probability of a false negative, i.e. the chance that a child 'at risk' is assigned to the low risk category, is minimized. A child will be assigned to the 'moderate risk' category if the child is born before 37 weeks gestation, has a Apgar score below seven, or has a congenital malformation. If the child also has a birth weight below 2,000 gram, the child will be assigned to the 'high risk' category. The other children will be assigned to the 'low risk' category. Based on figures reported by the PRN [12] and expert opinion it is expected that approximately 8% of the singletons will be assigned to the 'moderate risk' or 'high risk' category. All 'moderate risk' and 'high risk' singletons and a random selection of 10% of the remaining ('low risk') singletons will be included in the base sample. Based on figures reported by the PRN [12] and expert opinion it is expected that half of the twin children is born before 37 weeks gestation, have a low APGAR score or a congenital malformation. Approximately 40% of these twin children will also have a low birth weight (20% of all twin children) and will be assigned to the 'high risk' category. The other 60% of these twin children will have a birth weight above 2000 gram (30% of all twin children) and will be assigned to the 'moderate risk' category [12]. All other children (50% of all twin children) will be assigned to the 'low risk' category. All twin children of the 'high risk' category, a random selection of 30% of the 'moderate risk' category and a random selection of 10% of the 'low risk' category will be included in the base sample.

#### Empirical data collection up to the first year of life

Data with respect to the first year of life will be collected in children of the base sample. Based on figures reported by the PRN over 2004 [12] and expert opinion it is expected that approximately 1,278 children will be included in the base sample. The estimated number of children in each risk category is shown in figure 1.

Cost and outcome data up to the first year of life will be obtained from the hospital information systems of the relevant hospitals, existing registries and, if necessary, patient charts will be reviewed to obtain relevant cost and outcome data. The following registries will be used: 1) the PRN, which encompasses information regarding mother, pregnancy and the newborn; 2) the National Medical Registration (LMR), which registers medical and administrative data of all patients who have been admitted or have received outpatient treatment in Dutch hospitals; and 3) the Netherlands Twin registry (NTR), which follows a cohort 72,000 twins regarding pre- and perinatal information, health status, growth and motor development and behaviour [53,54]. As we intend to collect follow up data directly from hospital records, the use of data from the LMR will be optional. It is expected that data collection up to the first year of life will succeed in 90% of these children.

#### Empirical data collection from the first year of life up to the fifth year of life

Based on empirical collected costs and outcome data up to the first year of life, a subgroup of children will be selected for data collection up to the fifth year of life, referred to as the reduced sample. All children with resolved or persistent health problems, and a random sample of children without resolved or persistent health problems will be included in the reduced sample. Assuming that missing data are completely at random, resolved and persistent health problems are expected in 4.0% of the singletons and in 12.5% of the twin children. Approximately 488 children will be included in the reduced sample (see figure 1). Besides the data sources mentioned above, cost and outcome data up to the fifth year of life will also be gathered by means of a parent questionnaire. The questionnaire will consist of three parts, the first part referring to the past, the second part referring to the present and the third part referring to expectations regarding the future. The parent questionnaire will focus on health outcomes, HRQoL and costs.

#### Data estimation up to the 18th year of life

Costs and outcome data of the reduced sample up to the 18^th ^year of life will be estimated, based on 1) empirical costs and outcomes up to five years of life; 2) parent estimations by means of a questionnaire; and 4) a matched sample (n = 250) of 18-year old twin children, of whom NTR data referring to the past will be studied. The sample from the NTR will be chosen such that their past health at five years of age matches with the health status of the children of the reduced sample who will have an average age of five years. If empirical data are missing, data will be obtained by means of literature and/or expert opinion. Based on these data inputs, a prognostic model - the TwinSing model - will be developed.

### Outcome measures

Outcome measures are:

1. Health outcomes of the singleton and twin children, such as (perinatal) mortality, morbidity, perinatal outcomes (birth weight, prematurity, APGAR), malformations, developmental problems (psychomotor, cognitive and growth) and behavioural outcomes.

2. Health related quality of life of the singleton and twin children. HRQoL will be measured via proxy ratings by (one of) the parents by means of the EuroQol (EQ-5D) and the Health Utilities Index (HUI3). The EuroQol provides a simple descriptive profile, combining scores on mobility, self-care, daily activities, pain/discomfort and depression/anxiety into a single index value for health status and a VAS scale (0-100). Utility scores for the health states will be calculated by using the UK-tariff [55] and by using the Dutch EQ-5D tariff [56]. The reliability and validity of the EQ-5D has been established [57]. The proxy version of the EQ-5D has good construct validity and convergent validity [58]. The HUI3 will also be used as some studies have reported problems regarding speech development and cognitive problems. The HUI3 is a generic measure consisting of eight attributes (vision, hearing, speech, ambulation, dexterity, emotion, cognition and pain). Each attribute has five or six levels and 972,000 possible health states can be defined. The HUI3 has a multiplicative scoring function which was derived from the Standard Gamble (SG) and Visual Analogue Scale (VAS) in a random sample of the Canadian general population, resulting in possible utility scores ranging from -0.36 to 1.00 [59].

3. (Societal) costs, including resources consumed within the health care sector, such as NICU admissions, hospital admissions, diagnostics, treatments, surgical interventions, and special needs (e.g. physiotherapy, occupational therapy or speech therapy); resources consumed outside the health care sector, such as support, special education, school absence and institutional care; patient and family costs, such as out-of-pocket expenses, travel costs and time costs; and productivity costs.

### Data analysis

#### Empirical data

Health outcomes of the singleton and twin children, including mortality, perinatal outcomes, morbidity and developmental and behavioural problems will be analysed. Subsequently, health outcomes will be expressed in QALYs. QALYs will be calculated based on mortality and HRQoL data retrieved from both the EQ-5D and the HUI. Costs will be calculated by multiplying volumes of resource use with the cost price. Cost prices will be obtained from the Dutch manual of costing studies [60] and financial departments of the participating centres. For cost prices that are not readily available, such as institutional care, special education and school absence, cost price calculations will be performed.

Descriptive statistics will be used to describe the main characteristics and outcomes of IVF singleton and twin children. The base sample and the reduced sample are not representative of the Dutch IVF population as a consequence of the risk stratification and the random selection of subsamples of healthy children. Therefore, before analysing the data, the proportions of high, moderate and low risk singletons and twin children will be adjusted, to properly reflect the ratios in the original population.

Results will be presented with and without stratification for maternal age and parity. The issue of selection (information) bias will be investigated explicitly by comparing PRN data of children with (completely) missing data with PRN data of children included in the analysis.

#### Development TwinSing model

A prognostic model will be developed which estimates the short and long-term costs and health outcomes of IVF singletons and IVF twin children. The structure and modelling method of the TwinSing model will be determined in close cooperation with experts in the project team and the participating centres. We expect that a Markov model will best suit the data; however, the final decision regarding which modelling method to use will be made after data become available.

#### Short and long-term cost-effectiveness of SET strategies versus DET strategies

The TwinSing model will be linked to a short-term Markov model developed at the University Hospital Maastricht [32], that compared seven embryo transfer strategies varying from three cycles SET to three cycles DET and several combination strategies. Parameter values were based on data from a large randomised controlled trial in which patients who started their first IVF cycle in the Maastricht University Medical Centre were included.

The economic evaluation will be performed from both the societal and the health care perspective and the time horizon will be 18 years. Cost and benefits occurring after one year will be discounted according to national guidelines [61].

The ultimate model - in which the short-term Markov model and the TwinSing model have been integrated - will enable the estimation of the cost-effectiveness of SET strategies versus DET strategies, expressed as the additional costs per life year gained and the additional costs per QALY. The cost-effectiveness will be analysed using a short term time frame (1-year) and a long term time frame (5 and 18-year).

Uncertainty in the model will be investigated by one-way sensitivity analysis and probabilistic sensitivity analysis. One-way sensitivity analysis will be used to test the robustness of the results for (methodological) uncertainties (e.g. discount rate and health-related quality of life instrument used). Parameter uncertainty will be further tested using probabilistic sensitivity analysis. Distributions will be fitted for all parameters in the model, except fixed parameters. Beta distributions will be fitted for probabilities and gamma distributions will be fitted for costs. Net monetary benefits (NMB) will be calculated for each strategy and cost-effectiveness acceptability curves (CEACs) will be constructed. CEACs provide a graphical representation of the probability that a strategy is cost-effective for a range of ceiling ratios. Subsequently, the cost-effectiveness acceptability frontier (CEAF) will be established, to indicate the preferred strategy.

As most parents prefer a complete family instead of one child, a secondary analysis will be performed in which the cumulative pregnancy rates and live birth rates of subsequent IVF treatments (up to three) will be estimated. Doing so, the costs and outcomes of children of the second or third IVF treatment will be evaluated to estimate the cost-effectiveness of SET strategies versus DET strategies.

#### Value of information analysis

Inherent to modelling is that results are surrounded by an amount of uncertainty. Value of information analysis addresses the issue whether the most efficient embryo transfer strategy can be adopted based on the available knowledge after finishing this study or whether more evidence is required [62-65]. An expected value of perfect information (EVPI) analysis will be performed, which draws on the probabilistic model developed in this study. Subsequently partial EVPIs will be calculated to identify the parameters for which more accurate estimates are the most valuable and which should be the focus of future research.

### Ethical considerations

The study protocol has been reviewed and approved by the Medical Ethical Committee of the Maastricht University Medical Centre (MUMC). Parents will be informed in written format about the TwinSing study and are asked to return a signed informed consent form if they decide to participate in the parent questionnaire.

### Funding

A grant was obtained from the efficiency research programme, round 2009, of the Netherlands Organization for Health and Development (ZonMW).

## Discussion

Multiple pregnancies are regarded as a complication of IVF. To date, SET is more and more accepted as the solution for the high frequency of twin pregnancies after IVF. To support the claim that twin pregnancies are a complication of IVF, the long-term costs and outcomes of twins and singletons should be determined. To our knowledge this is the first study on both long-term (societal) costs and health outcomes of IVF twins and singletons, allowing us to calculate the long-term cost-effectiveness of several (combinations of) SET and/or DET strategies.

This study will use QALYs as outcome measure, which will enhance comparison with other health care programs and the interpretation of the ICERs. Furthermore, this study will be conducted from the societal perspective and this includes not only hospital costs, but also productivity, patient and family costs. Most previous studies focused on hospital costs only, neglecting costs outside the healthcare sector. Analyses will also be performed from the healthcare perspective.

Another strength of this study protocol is its efficient design. The larger part of the health problems and associated costs will occur in a relatively small group of children. The study is set up such that data collection focuses on these children. Data collection regarding the first year of life is focused on children at high risk of health problems due to a prematurity, a low Apgar score, a congenital malformation or a low birth weight. Data collection regarding the second to fifth year of life is focused on children who experienced health problems during their first life year. Additional advantage is that, due to the decreasing number of participants making up the sample size in later stages of the study, a particularly high level of detail can be reached in the data collection.

Although this study will apply the commonly used QALY as measure of outcome, a number of potential problems are to be expected. The first question is whether the QALY concept is suitable to value QALYs '*generated' *or '*created'*, instead of QALYs *gained*, for which the QALY framework was originally developed. Furthermore, a twin consists of two live-born children. An interesting issue in using QALYs as outcome measure is that a healthy twin will theoretically generate twice as much QALYs as a healthy singleton, which is the total opposite compared to how twins have been valued in cost-effectiveness analyses thus far. As twins are more common among woman receiving DET, including the QALYs of both twin children in the cost-effectiveness analysis will have considerable impact on the ICERs [32]. Furthermore, it can be expected that parents with a single child after SET, will more often undergo subsequent IVF treatment(s) than parents with a twin after DET. As a result, the long-term cost-effectiveness analysis including only the first IVF treatment is of limited value. It does not reflect the real world situation in which parents can opt for up to three IVF treatments. Our secondary analysis including subsequent IVF treatments will take account of this imbalance.

Moreover, when using QALYs, an interesting question is furthermore whether the QALYs of parents and siblings should be taken into account as well. For example, the birth of a (handicapped) child may have a considerable impact on family members. It should be considered, if and how QALYs of the parent and family members should be included in the analysis.

## Conclusion

This study will provide information on the short and long-term incremental (societal costs) and health outcomes of IVF twin children compared with IVF singletons. Subsequently, it will provide further evidence regarding whether SET strategies are more efficient than DET strategies. Results of this study will be of interest for patients, clinicians, health care policymakers and insurers, both nationally and internationally.

## Competing interests

The authors declare that they have no competing interests.

## Authors' contributions

All authors read and approved the final version of the manuscript for publication. MMJH, the PhD-researcher of the TwinSing study, drafted the study protocol and final manuscript. CDD is the project leader of the TwinSing study. She conceived the study and its design, secured its funding, is providing leadership and coordination for the research study, and provided substantial commentary to the final submitted manuscript. GJB, JCMD, JLHE, MAHBMH and JLS participated in designing the study, securing grant funding, and provided critical commentary to the final submitted manuscript. All other authors are involved in optimization of the study protocol and critically revised it for important intellectual content.

## Pre-publication history

The pre-publication history for this paper can be accessed here:

http://www.biomedcentral.com/1471-2431/10/75/prepub
